# Supplementary material for: MORF and MOZ acetyltransferases target unmethylated CpG islands through the winged helix domain
Source: Nat Commun. 2023 Feb 8;14:697. doi: 10.1038/s41467-023-36368-5 (PMC9908889; doi:10.1038/s41467-023-36368-5)
Supplement: Supplementary file 1 — Supplementary Information [file 41467_2023_36368_MOESM1_ESM.pdf]

Supplementary Information

MORF and MOZ acetyltransferases target unmethylated CpG islands through the winged helix domain

Dustin C. Becht, et al.

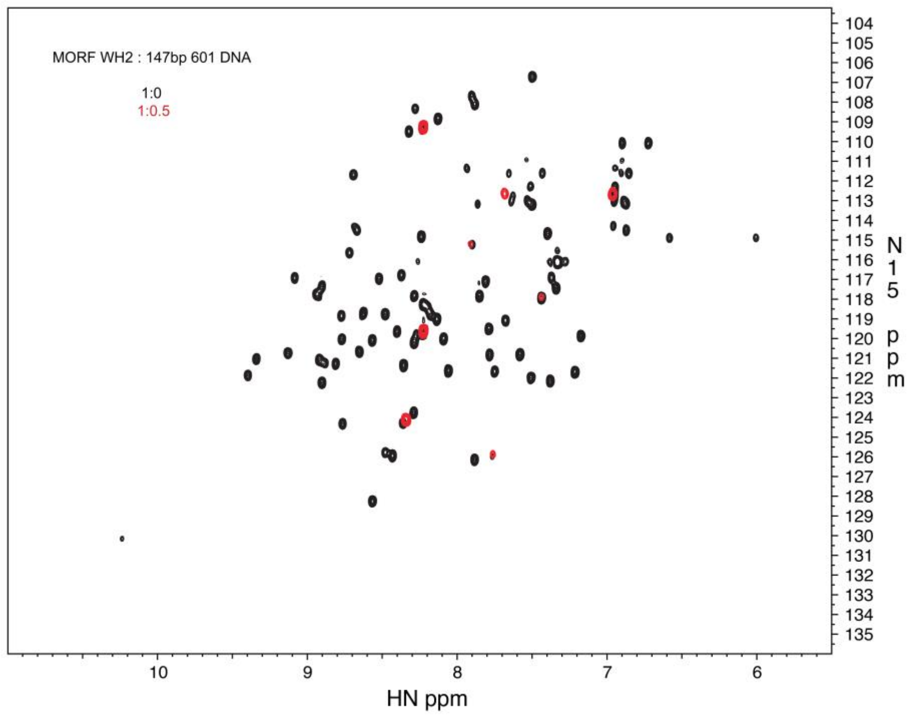

Supplementary Figure 1. Overlay of $^{1}H,^{15}N$ HSQC spectra of $MORF_{WH2}$ in the absence (black) and presence of 601 DNA (red). Related to Figure 1.

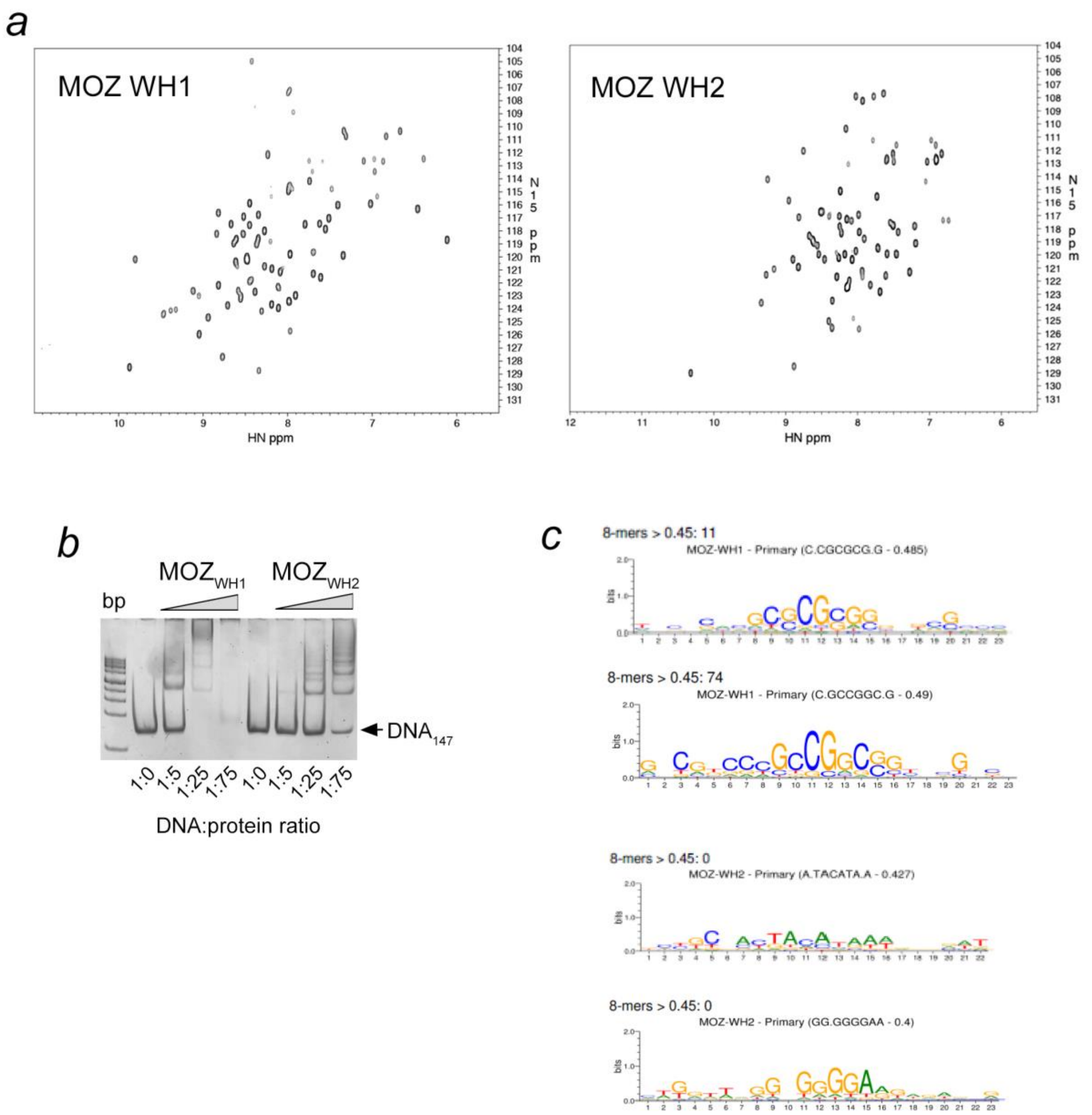

Supplementary Figure 2. (a) ^{1}H,^{15}N HSQC spectra of MOZ_{WH1} and MOZ_{WH2}. (b) EMSA of 147bp 601 DNA in the presence of increasing amounts of MOZ_{WH1} and MOZ_{WH2}. DNA:protein ratio is shown below the gel image. (c) Analysis of DNA binding selectivity of MOZ_{WH1} and MOZ_{WH2} in universal oligonucleotide PBM arrays. Related to Figures 1 and 3. Source data are provided as a Source Data file.

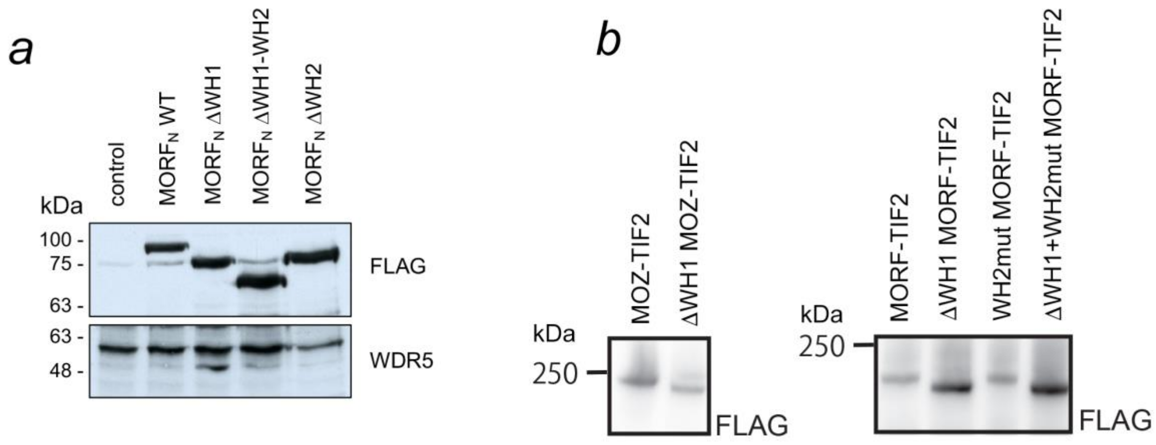

Supplementary Figure 3. Western-Blot showing expression of the indicated MORF and MOZ constructs. (a) FLAG-MORF$_{N1-716}$ WT, ΔWH1, ΔWH2 and ΔWH1-WH2 in the respective K562 cell lines used for ChIP experiments. WDR5 signal is used as loading control. (b) Protein expression of FLAG-tagged MOZ/MORF-TIF2 fusion constructs in plat-E cells. The whole cell extracts were analyzed by Western-Blotting using anti-FLAG antibody. Related to Figures 1 and 6. Source data are provided as a Source Data file.

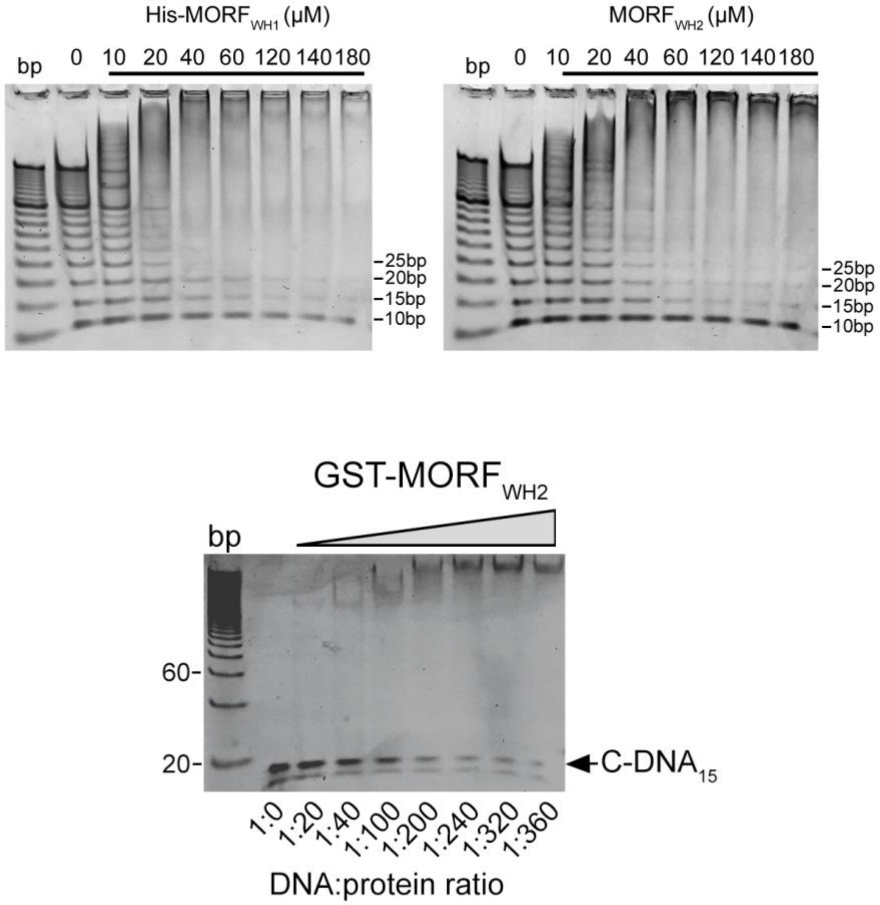

Supplementary Figure 4. EMSAs of a 10bp to 100bp DNA ladder (top) or a 15bp C-rich dsDNA (C-DNA$_{15}$) (bottom) in the presence of increasing amounts of indicated MORF$_{WH1}$ and MORF$_{WH2}$. Related to Figures 2 and 3. Source data are provided as a Source Data file.

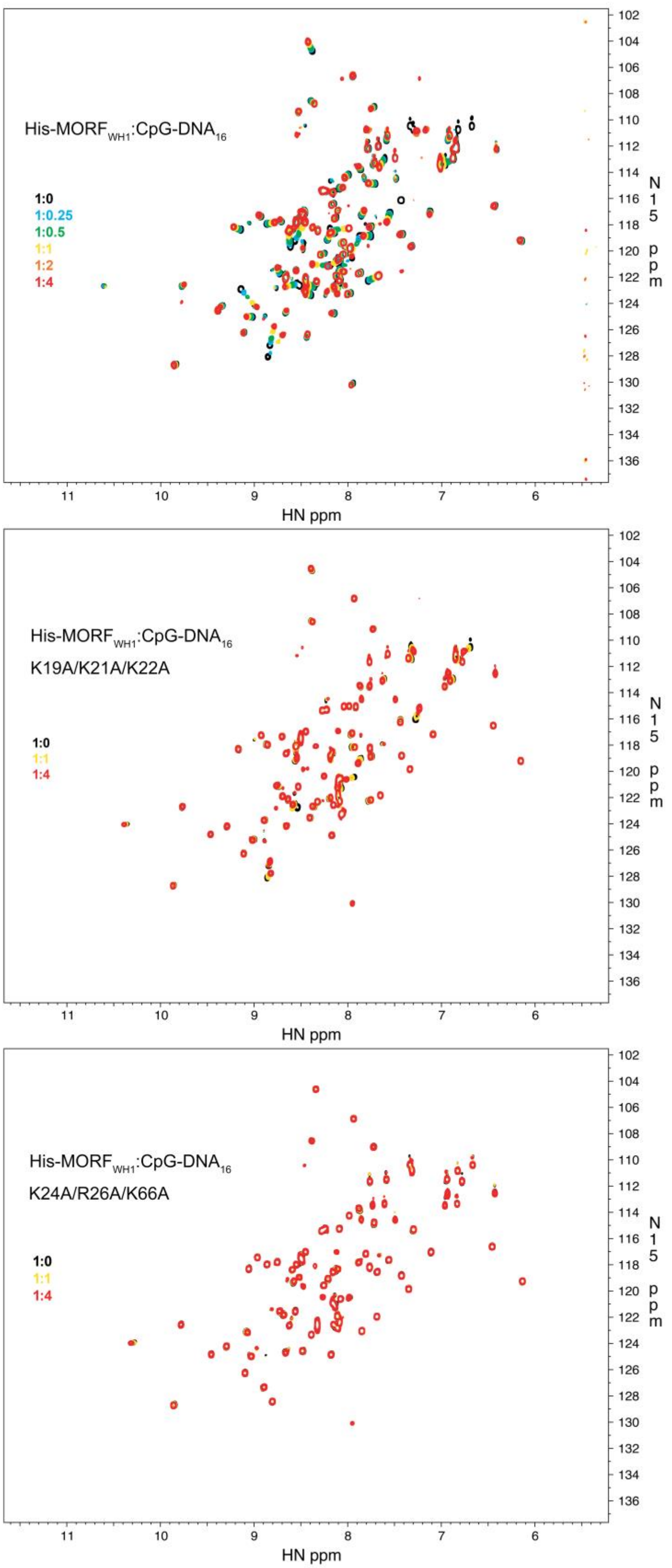

Supplementary Figure 5. Overlay of $^{1}H,^{15}N$ HSQC spectra of ^{15}N-labeled His-MORF$_{WH1}$, WT or K19A/K21A/K22A and K24A/R26A/K66A mutants in the presence of increasing amount of CpG-DNA$_{16}$. Spectra are color coded according to the protein:DNA molar ratio. Related to Figure 3.

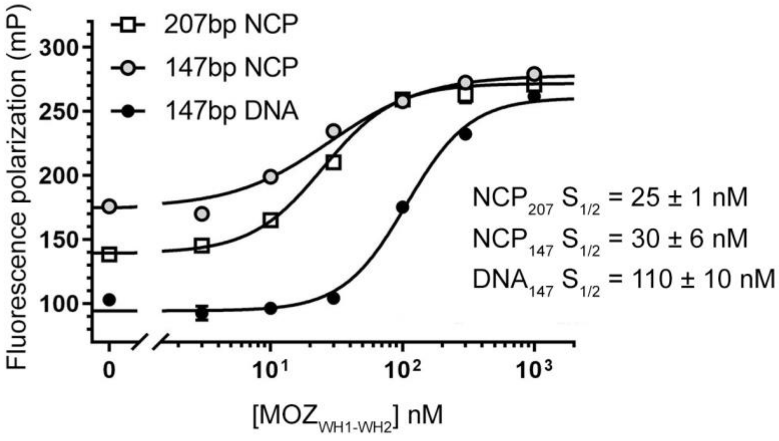

Supplementary Figure 6. Binding curves for the interactions of $MOZ_{WH1-WH2}$ with NCP_{207}, NCP_{147} and DNA_{147} as measured by fluorescence polarization. Data represent mean ± SD of three independent experiments. n=3 Related to Figure 7. Source data are provided as a Source Data file.

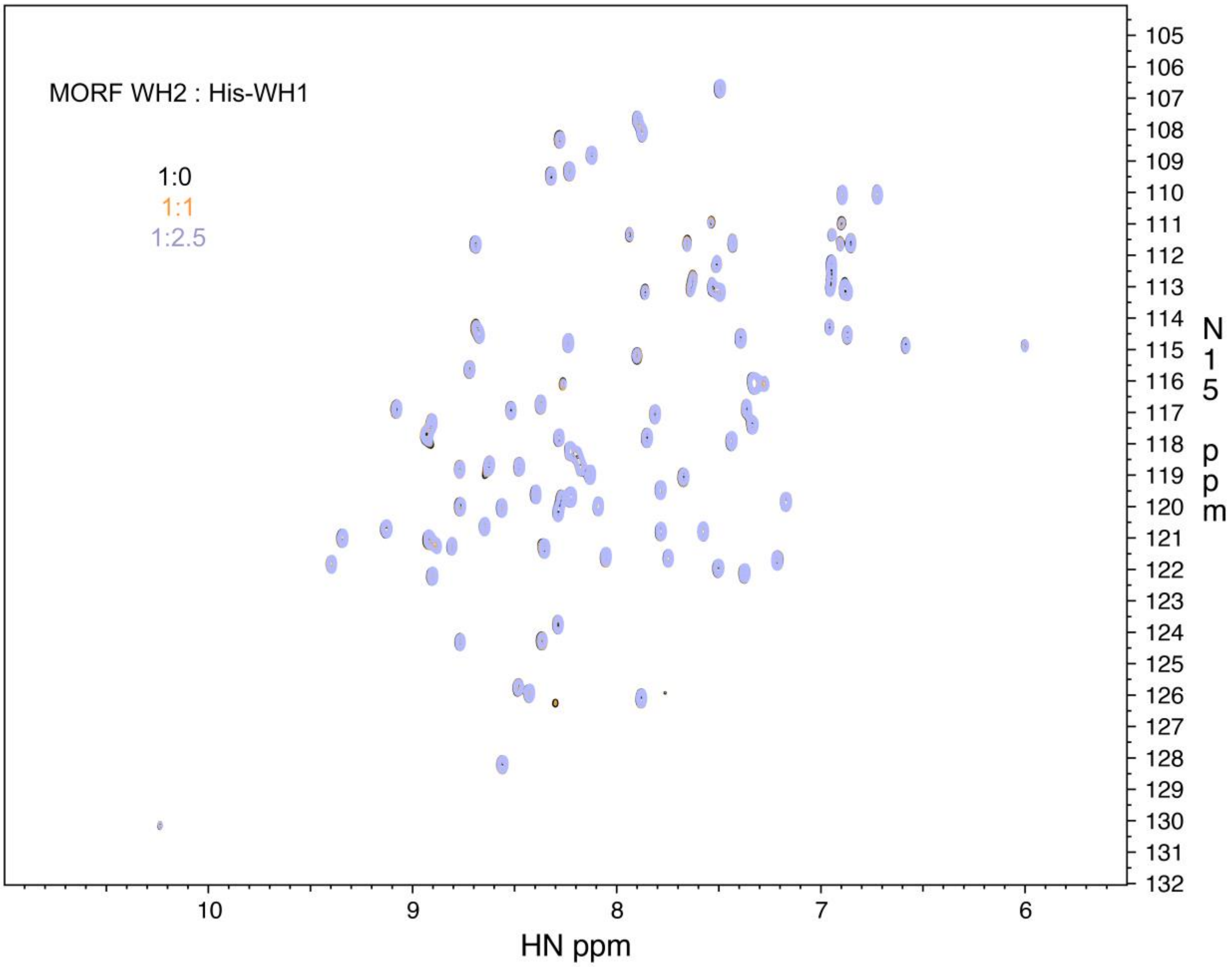

Supplementary Figure 7. Overlay of $^{1}H,^{15}N$ HSQC spectra of ^{15}N-labeled $MORF_{WH2}$ in the presence of increasing amount of unlabeled His-$MORF_{WH1}$. Spectra are color coded according to the protein:ligand molar ratio. Related to Figure 7.

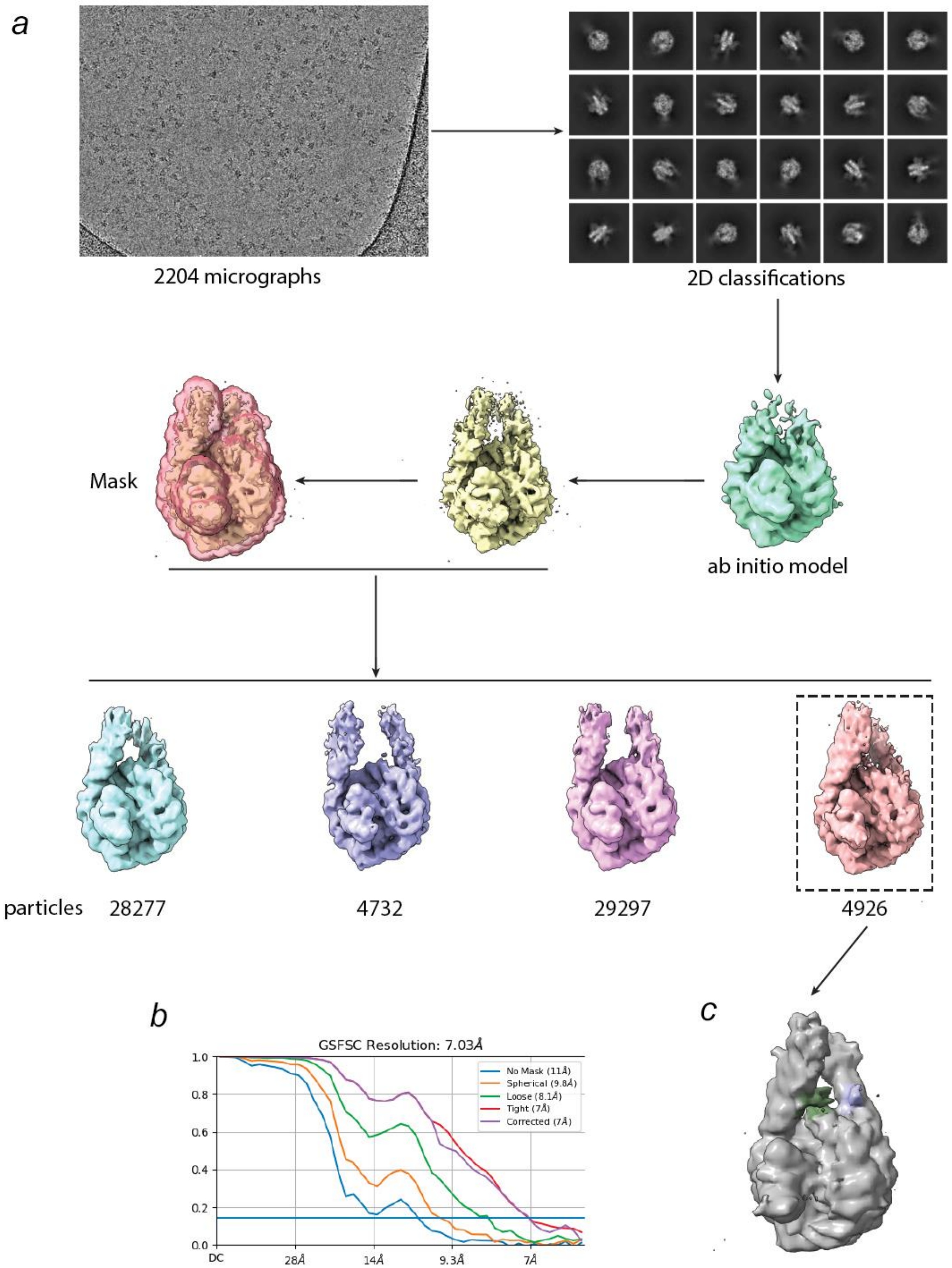

Supplementary Figure 8. Cryo-EM reconstruction of $MORF_{WH1\text{-}WH2}$-Nucleosome-scFv complex. (a) Workflow of the cryo-EM image processing. (b) Fourier Shell Correlations (FSC) of the reconstruction. Gold-standard cut-off (FSC=0.143) marked with a blue line. (c) Final cryo-EM map of the $MORF_{WH1\text{-}WH2}$-nucleosome-scFv complex, the density near the dyad region is colored in green and light blue. Related to Figure 8.

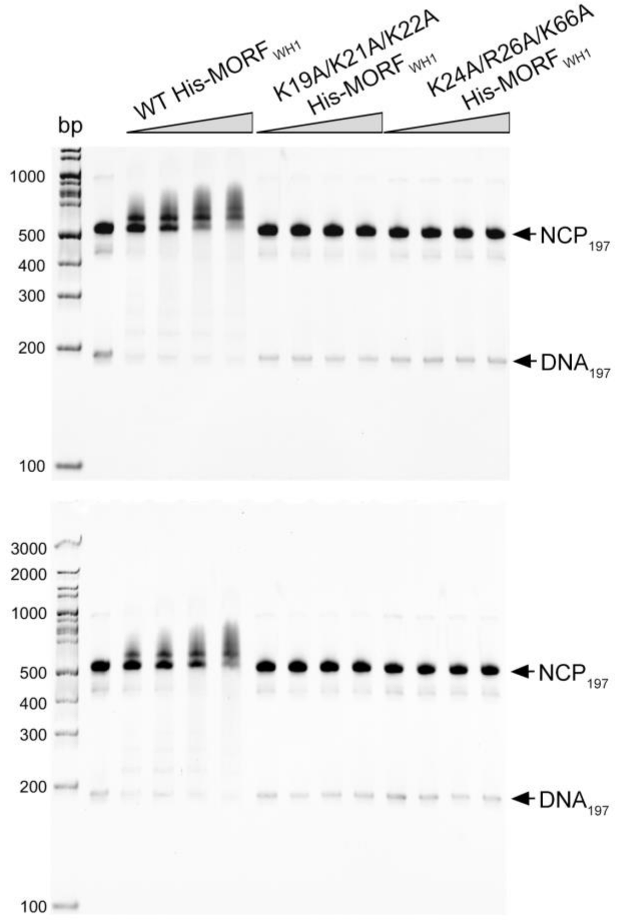

Supplementary Figure 9. Two replicates of EMSA of 197 bp NCP (NCP_{197}) in the presence of increasing amounts of indicated WT or mutated His-$MORF_{WH1}$. Related to Figure 8. Source data are provided as a Source Data file.

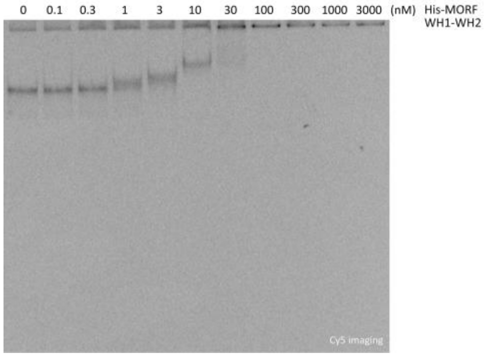

Supplementary Figure 10. EMSA of the Cy3-Cy5 labeled NCP_{273} in the presence of increasing amounts of His-$MORF_{WH1\text{-}WH2}$. Related to Figure 8. Source data are provided as a Source Data file.

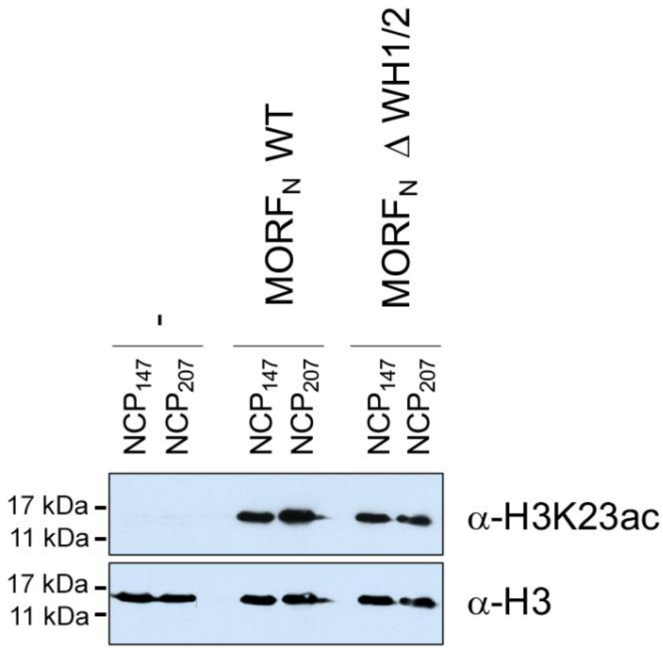

Supplementary Figure 11. *In vitro* HAT assays using WT and mutated MORF$_N$ complexes purified from K562 cells on NCP w/o DNA linkers (NCP$_{147}$) and NCP with the linkers (NCP$_{207}$). Related to Figure 9. Source data are provided as a Source Data file.

Supplementary Table 1. NMR and refinement statistics for the $MORF_{WH2}$ structures. Related to Figure 2.

	$MORF_{WH2}$
**NMR distance and dihedral constraints**	
Distance constraints	
Total NOE	1603
Intra-residue	600
Inter-residue	
Sequential ($\|i-j\| = 1$)	390
Medium-range ($\|i-j\| \leq 4$)	303
Long-range ($\|i-j\| \geq 5$)	280
Hydrogen bonds	40
Total dihedral angle restraints	
ϕ	54
ψ	54
**Structure statistics**	
Violations (mean and s.d.)	
Distance constraints (Å)	0.054 ± 0.001
Dihedral angle constraints (º)	0.842 ± 0.078
Number of distance violation (> 0.35 Å)	0
Number of angle violation (> 10º)	0
Deviations from idealized geometry	
Bond lengths (Å)	0.003 ± 0.000
Bond angles (º)	0.461 ± 0.023
Impropers (º)	0.333 ± 0.017
Average pairwise r.m.s. deviation** (Å)	
Heavy	2.233 ± 0.655
Backbone	1.818 ± 0.743
Heavy (107-118,126-135,140-144,148-162,165-168,171-174)	0.895 ± 0.094
Backbone (107-118,126-135,140-144,148-162,165-168,171-174)	0.309 ± 0.068

**Pairwise r.m.s. deviation to lowest energy structure was calculated among 15 refined structures. Residues 100-182 of MORF were used.

Supplementary Table 2. Taqman probes. Related to Figure 6.

Oligonucleotides		
Taqman probe for murine *Gapdh*	Life Technologies	Mm99999915_g1
Taqman probe for murine *Hoxa9*	Life Technologies	Mm00439364_m1

Supplementary Table 3. Custom qPCR probe/primer sequences. Related to Figure 6.

Target name	Forward	Reverse	Reporter
CD4- pre-TSS	TGTCCGAGCAAGGGATGATATTG	CCAAGTCACTCTGCACTACCA	ACTGCCACCATGCCAAT
CD4-TSS	GCAGCGGGCAAGAAAGAC	ACGTTCCCCTCCCTCTCA	CCGAGTCTGACCACCTTAC
CD4- post-TSS	GGACTCACTGTCCCTCCTGAA	CTTCAAGGCCATGAGGTCTCA	TCAGCCTTTCCGCCCTC
MYC pre-TSS	GCGGTATCTGCTGCTTTGG	GCATTATGTATGCACAGCTATCTGGAT	CTGGGTGGAAGGTATCC
MYC-TSS	CCGGCTAGGGTGGAAGAG	GAGGCGAAGCCCCCTATTC	CAGGACGCCCGCAGCG
MYC post-TSS	GGGTAGGCGCAGGCA	GGTTTTTCCAAGTCAACGATTCCA	ATGTGTCCGATTCTCC
HOXA9 pre-TSS	TGGCTGCTTTTTTATGGCTTCAATT	CCGCGTGCGAGTGC	CCCCTCACATAAAATT
HOXA9-TSS	TCACCACCACCCCTACGT	GCAAGCCCGCGAAGGA	CAGGAGCGCATGTACC
HOXA9 post-TSS	AGTGGCGGCGTAAATCCT	TGATCACGTCTGTGGCTTATTTGAA	CCCGCAGCCTCATC
CDKN2C-pre-TSS	CTCCACAACCGTCTTAAATAACAAACC	GCGGGCTTGAGTCTGTGA	CAGCTGCCCCAATTC
CDKN2C-TSS	GGCGGCTGCCCTGT	CCCGGTGCCACTTTGC	CTGTGCCCCTTTGCTG
CDKN2C-post-TSS	CTGTGGAGTCGTCAGAATTCTTCAT	CGATTCACACGTGATTATTCAGCAAA	CCTCGCCTCGCTTTT

Supplementary Table 4. Accession numbers of the NGS data. Related to Figures 4-6.

Sample name	DRA accession number	Sample ID	GEA accession number
Input gDNA (CIRA-seq)	DRA008734	SAMD00180208	E-GEAD-324
Unmethylated CpGs (CIRA-seq)	DRA008734	SAMD00180209	E-GEAD-324
Input chromatin (ChIP)	DRA012473	SAMD00393839	E-GEAD-446
MOZ$_{FL}$ (ChIP)	DRA008732	SAMD00180127	E-GEAD-322
MEAF6 (ChIP)	DRA012473	SAMD00393844	E-GEAD-446
ING4 (ChIP)	DRA012473	SAMD00393843	E-GEAD-446
H3K14ac (ChIP)	DRA014291	SAMD00495580	E-GEAD-497
RNAP2 non-P (ChIP)	DRA012473	SAMD00393846	E-GEAD-446
RNAP2 Ser5-P (ChIP)	DRA012473	SAMD00393847	E-GEAD-446
Vector-FLAG (ChIP)	DRA014290	SAMD00495574	E-GEAD-498
MOZ$_{WH1}$-FLAG rep1(ChIP)	DRA014290	SAMD00495575	E-GEAD-498
MOZ$_{WH1}$-FLAG rep2 (ChIP)	DRA014290	SAMD00495576	E-GEAD-498
MOZ$_{WH1-WH2-DPF}$-FLAG (ChIP)	DRA014290	SAMD00495578	E-GEAD-498
MOZ$_{WH2-DPF}$-FLAG rep1 (ChIP)	DRA014290	SAMD00495577	E-GEAD-498
MOZ-TIF2-FLAG (ChIP)	DRA010562	SAMD00237834	E-GEAD-381
Vector-FLAG rep2 (ChIP)	DRA015383	SAMD00567463	E-GEAD-584
MOZ$_{WH1}$-FLAG rep3 (ChIP)	DRA015383	SAMD00567464	E-GEAD-584
MORF$_{WH1}$-FLAG rep1(ChIP)	DRA015383	SAMD00567465	E-GEAD-584
MORF$_{WH1}$-FLAG rep2(ChIP)	DRA015383	SAMD00567466	E-GEAD-584

Supplementary Table 5. Cryo-EM data collection statistics. Related to Figure 8.

	$MORF_{WH1-WH2}$ - nucleosome - scFv (EMDB-27243)
**Data collection and processing**	
Magnification	56,000
Voltage (kV)	200
Electron exposure (e–/Å^2)	43.2
Defocus range (μm)	0.8-1.8
Pixel size (Å)	0.91
Symmetry imposed	C1
Initial particle images (no.)	219,800
Final particle images (no.)	4,926
Map resolution (Å)	7.03
FSC threshold	0.143
Map resolution range (Å)	5.8-20
