# Supplementary material for: MORF and MOZ acetyltransferases target unmethylated CpG islands through the winged helix domain
Source: Nat Commun. 2023 Feb 8;14:697. doi: 10.1038/s41467-023-36368-5 (PMC9908889; doi:10.1038/s41467-023-36368-5)
Supplement: Supplementary file 3 — Reporting Summary [file 41467_2023_36368_MOESM3_ESM.pdf]

nature portfolio

Corresponding author(s): Tatiana Kutateladze

Last updated by author(s): Jan 18, 2023

Reporting Summary

Nature Portfolio wishes to improve the reproducibility of the work that we publish. This form provides structure for consistency and transparency in reporting. For further information on Nature Portfolio policies, see our Editorial Policies and the Editorial Policy Checklist.

Statistics

For all statistical analyses, confirm that the following items are present in the figure legend, table legend, main text, or Methods section.

n/a	Confirmed	
☐	☒	The exact sample size (*n*) for each experimental group/condition, given as a discrete number and unit of measurement
☐	☒	A statement on whether measurements were taken from distinct samples or whether the same sample was measured repeatedly
☐	☒	The statistical test(s) used AND whether they are one- or two-sided<br>*Only common tests should be described solely by name; describe more complex techniques in the Methods section.*
☒	☐	A description of all covariates tested
☒	☐	A description of any assumptions or corrections, such as tests of normality and adjustment for multiple comparisons
☐	☒	A full description of the statistical parameters including central tendency (e.g. means) or other basic estimates (e.g. regression coefficient) AND variation (e.g. standard deviation) or associated estimates of uncertainty (e.g. confidence intervals)
☐	☒	For null hypothesis testing, the test statistic (e.g. *F*, *t*, *r*) with confidence intervals, effect sizes, degrees of freedom and *P* value noted<br>*Give P values as exact values whenever suitable.*
☒	☐	For Bayesian analysis, information on the choice of priors and Markov chain Monte Carlo settings
☒	☐	For hierarchical and complex designs, identification of the appropriate level for tests and full reporting of outcomes
☐	☒	Estimates of effect sizes (e.g. Cohen's *d*, Pearson's *r*), indicating how they were calculated

Our web collection on statistics for biologists contains articles on many of the points above.

Software and code

Policy information about availability of computer code

Data collection	NMR experiments were carried out at 298K on Varian INOVA 600 and 900 MHz spectrometers at the UC Denver NMR Core facility. Cryo-EM images were collected on a Talos Arctica microscope at 200kV with a Gatan K3 Summit direct detection camera at the NCI-Frederick cryo-EM Facility.
Data analysis	NMR data were analyzed using NMRPipe vLinux, NMRDraw v11.2, CcpNmr v3.0, XPLOR-NIH v3.4, PROCHECK-NMR v3.5.4 and other software listed in the Method section. Cryo-EM data were processed using cryoSPARC v3 software package listed in the Method section. Cryo-EM maps were illustrated using UCSF ChimeraX (https://www.rbvi.ucsf.edu/chimerax). All statistical data were analyzed with Prism 8 software (GraphPad software) and Microsoft Excel v16.68.

For manuscripts utilizing custom algorithms or software that are central to the research but not yet described in published literature, software must be made available to editors and reviewers. We strongly encourage code deposition in a community repository (e.g. GitHub). See the Nature Portfolio guidelines for submitting code & software for further information.

Data

Policy information about availability of data

All manuscripts must include a data availability statement. This statement should provide the following information, where applicable:

- Accession codes, unique identifiers, or web links for publicly available datasets
- A description of any restrictions on data availability
- For clinical datasets or third party data, please ensure that the statement adheres to our policy

The data that support this study are available from the corresponding authors upon reasonable request. Coordinates and structure factors have been deposited in the Protein Data Bank under accession code 8E4V [http://doi.org/10.2210/pdb8E4V/pdb]. NMR data have been deposited in the Biological Magnetic Resonance Bank under accession number 31040 [https://bmrb.io/data_library/summary/index.php?bmrbId=31040]. Cryo-EM map of the MORFWH1-WH2 and 197 bp nucleosome complex has been deposited in the Electron Microscopy Data Bank under accession number EMD-27243 [https://www.emdataresource.org/EMD-27243]. ChIP-seq and CIRA-seq data have been deposited to the DDBJ (DNA Data Bank of Japan) Sequence Read Archive as fastq files and as WIG files under accession numbers DRA008734, DRA012473, DRA008732, DRA014291, DRA014290, DRA010562, DRA015383, E-GEAD-324, E-GEAD-446, E-GEAD-322, E-GEAD-497, E-GEAD-498, E-GEAD-381 and E-GEAD-584 [https://ddbj.nig.ac.jp/public/ddbj_database/dra/fastq/] and [https://ddbj.nig.ac.jp/public/ddbj_database/gea/experiment/E-GEAD-000/] (Supplementary Table 4). The mass spec data have been deposited to the PRIDE database [http://www.ebi.ac.uk/pride] under accession number PXD036192. Source Data are provided with this paper.

Human research participants

Policy information about studies involving human research participants and Sex and Gender in Research.

Reporting on sex and gender	n/a
Population characteristics	n/a
Recruitment	n/a
Ethics oversight	n/a

Note that full information on the approval of the study protocol must also be provided in the manuscript.

Field-specific reporting

Please select the one below that is the best fit for your research. If you are not sure, read the appropriate sections before making your selection.

☒ Life sciences ☐ Behavioural & social sciences ☐ Ecological, evolutionary & environmental sciences

For a reference copy of the document with all sections, see nature.com/documents/nr-reporting-summary-flat.pdf

Life sciences study design

All studies must disclose on these points even when the disclosure is negative.

Sample size	The chosen sample sizes are based on the numbers used for previous publications and are present in relevant figure legends
Data exclusions	no data exclusions
Replication	present in relevant figure legends
Randomization	Randomization was not a relevant feature as we applied a uniform set of techniques.
Blinding	Blinding was not a relevant feature as we applied a uniform set of techniques and tests were repeated two or more times by different individuals.

Reporting for specific materials, systems and methods

We require information from authors about some types of materials, experimental systems and methods used in many studies. Here, indicate whether each material, system or method listed is relevant to your study. If you are not sure if a list item applies to your research, read the appropriate section before selecting a response.

Materials & experimental systems

n/a	Involved in the study
☐	☒ Antibodies
☐	☒ Eukaryotic cell lines
☒	☐ Palaeontology and archaeology
☐	☒ Animals and other organisms
☒	☐ Clinical data
☒	☐ Dual use research of concern

Methods

n/a	Involved in the study
☐	☒ ChIP-seq
☒	☐ Flow cytometry
☒	☐ MRI-based neuroimaging

Antibodies

Antibodies used	Antibodies used for assays: 3 µg anti-FLAG M2 (F1804, Sigma), 1 µg anti-H3 (ab1791, Abcam), anti-H3K14ac (07-353, Upstate) and anti-H3K23ac (07-355, Upstate), and Alexa488 conjugated anti-GST antibody (Invitrogen A-11131, 1:40 dilution). Anti-FLAG M2 conjugated to horseradish peroxidase (A8592, Sigma) was used at 1:10,000 dilution. The following antibodies were used in in vitro HAT assays: anti-H3K23ac (Upstate, 07-355, 1:1,000 dilution) and anti-H3 (Abcam, ab1791, 1:20,000 dilution). For purification of native MORF complexes, anti-FLAG M2 (Sigma, F1804, 1:10,000 dilution) and anti-WDR5 (a gift from Edwin Smith, 1:1,000 dilution) antibodies were used.
Validation	All antibodies validation are available on the manufacturers' websites.

Eukaryotic cell lines

Policy information about cell lines and Sex and Gender in Research

Cell line source(s)	HEK293T cells were purchased from ATCC. Isogenic K562 cell lines expressing 3xFlag2xStrep-tagged MORF constructs were generated by integration at the AAVS1 safe harbor locus after DSB induction and recombination targeted by co-transfection with a ZFN expression plasmid.
Authentication	HEK293T cells were authenticated by the manufacturer.
Mycoplasma contamination	HEK293T cells were tested negative for mycoplasma contamination.
Commonly misidentified lines (See ICLAC register)	No commonly misidentified cell lines were used in the study.

Animals and other research organisms

Policy information about studies involving animals; ARRIVE guidelines recommended for reporting animal research, and Sex and Gender in Research

Laboratory animals	Mouse: C57BL/6JJcl CLEA Japan, Inc. C57BL/6JJcl
Wild animals	N/A
Reporting on sex	Female mice were used.
Field-collected samples	N/A
Ethics oversight	The experiment was approved by the institutional review board at the National Cancer Center and performed by abiding by ethical regulations

Note that full information on the approval of the study protocol must also be provided in the manuscript.

ChIP-seq

Data deposition

☒ Confirm that both raw and final processed data have been deposited in a public database such as GEO.

☒ Confirm that you have deposited or provided access to graph files (e.g. BED files) for the called peaks.

Data access links *May remain private before publication.*	ChIP-seq and CIRA-seq data have been deposited to the DDBJ (DNA Data Bank of Japan) Sequence Read Archive under accession numbers and sample IDs listed in Suppl. Table 4.<br>https://ddbj.nig.ac.jp/public/ddbj_database/dra/fastq/DRA008/DRA008732/<br>https://ddbj.nig.ac.jp/public/ddbj_database/dra/fastq/DRA008/DRA008734/<br>https://ddbj.nig.ac.jp/public/ddbj_database/dra/fastq/DRA010/DRA010562/

https://ddbj.nig.ac.jp/public/ddbj_database/dra/fastq/DRA012/DRA012473/
https://ddbj.nig.ac.jp/public/ddbj_database/dra/fastq/DRA014/DRA014290/
https://ddbj.nig.ac.jp/public/ddbj_database/dra/fastq/DRA014/DRA014291/
https://ddbj.nig.ac.jp/public/ddbj_database/dra/fastq/DRA015/DRA015383/
https://ddbj.nig.ac.jp/public/ddbj_database/gea/experiment/E-GEAD-000/E-GEAD-322/
https://ddbj.nig.ac.jp/public/ddbj_database/gea/experiment/E-GEAD-000/E-GEAD-324/
https://ddbj.nig.ac.jp/public/ddbj_database/gea/experiment/E-GEAD-000/E-GEAD-381/
https://ddbj.nig.ac.jp/public/ddbj_database/gea/experiment/E-GEAD-000/E-GEAD-446/
https://ddbj.nig.ac.jp/public/ddbj_database/gea/experiment/E-GEAD-000/E-GEAD-498/
https://ddbj.nig.ac.jp/public/ddbj_database/gea/experiment/E-GEAD-000/E-GEAD-497/
https://ddbj.nig.ac.jp/public/ddbj_database/gea/experiment/E-GEAD-000/E-GEAD-584/

Files in database submission

accession numbers and sample IDs are listed above and in Suppl. Table 4.

Genome browser session
(e.g. UCSC)

no longer applicable

Methodology

Replicates

Most of the ChIP-Seq data are from one experiment. ChIP analysis of the chromatin for FLAG-tagged MOZ WH1 was done in three biological replicates and MORF WH1 was done in two biological replicates.

Sequencing depth

Total number of reads, Uniquely mapped reads, Length of reads, Paired or single-end
Input gDNA (CIRA-seq) T_gDNA_aln…., 22383680, 18035983, 50 single-end
Unmethylated CpGs (CIRA-seq)T0207_unmethyl8_conc…, 23009042, 10707998, 50 single-end
Input chromatin (ChIP)21T0226-IN, 28759722, 25285507, 50 single-end
MOZFL (ChIP)T1117_MYST3…, 31089812, 19713471, 50 single-end
MEAF6 (ChIP) 21T0226-MEAF6…., 25985182, 22782710, 50 single-end
H3K14ac (ChIP) 21T0226-H3K14ac…, 30519269, 26708743, 50 single-end
RNAP2 non-P (ChIP) 21T0226-RNAP2_non-P, 34258133, 30680875, 50 single-end
RNAP2 Ser5-P (ChIP) 21T0226-RNAP2_Ser5-P, 30850929, 27719450, 50 single-end
Vector-FLAG (ChIP)T0328-V-rep1, 59190220, 43621379 100 single-end
MOZWH1-FLAG rep1(ChIP) T0328-f1-84-rep1, 61448755, 46635003, 100 single-end
MOZWH1-FLAG rep2 (ChIP) T0328-f1-84-rep2, 66558552, 51247745, 100 single-end
MOZWH1-WH2-DPF-FLAG (ChIP) rep1T0328-f1-487-rep1, 65734657, 49569168, 100 single-end
MOZWH2-DPF-FLAG rep1 (ChIP) T0328-f1-85-487-rep1, 82897042, 62259726, 100 single-end
MOZ-TIF2-FLAG (ChIP) T0513-fMOZ-TIF2-M2, 27292467, 22770986, 50 single-end
MEAF6 (ChIP) 21T0226-ING4, 30423675, 26093823, 50 single-end
Input chromatin (ChIP)T0328-V-rep1, 61777985, 47687124, 100 single-end
Vector-FLAG rep2 (ChIP), 48793968, 36354790, 100 single-end
MOZWH1-FLAG rep3 (ChIP), 79260816, 61115830, 100 single-end
MORFWH1-FLAG rep1 (ChIP) , 61609858, 49997402, 100 single-end
MORFWH1-FLAG rep2 (ChIP), 63423053, 51668662, 100 single-end

Antibodies

FLAG Sigma-Aldrich F3165/M2 (1:400), MOZ active motif 39868 (1:400), MEAF6 STJ 116836 (1:400), ING4 Abcam 108621 (1:400), Histone H3K14ac Abcam ab52946 (1:400), RNAP2 non-P Abcam 8WG16/ab817 (1:400), RNAP2 Ser5-P Millipore CTD4H8/05-623 (1:400)

Peak calling parameters

Peak calling was not performed in this study.

Data quality

All sequenced reads that cleared the illumina's pass filter were mapped to human genome assembly hg19 using BWA 0.7.5.

Software

The alignment tags were counted and ppm was calculated every 25 bp from TSS and the ChIP signal distribution was plotted using NGSplot 2.61. The BAM alignment was converted to the bBigWig coverage files using bam2wig 1.6. and wigToBigWig.
